# Tethering of the spinal cord in cervical region in adult male patient

**DOI:** 10.11604/pamj.2021.40.20.31405

**Published:** 2021-09-08

**Authors:** Neha Vinay Chitale, Mitushi Kishorrao Deshmukh

**Affiliations:** 1 Department of Musculoskeletal Physiotherapy, Ravi Nair Physiotherapy College, Datta Meghe Institute of Medical Sciences, Wardha, Maharashtra, India,; 2 Department of Musculoskeletal Physiotherapy, Ravi Nair Physiotherapy College, Sawangi (M), Wardha, Maharashtra, India

**Keywords:** Tethering of spine, myelomeningocele, rehabilitation

## Image in medicine

We report a case of a 31-year-old male, came to the medicine department with complains of pain and numbness in upper limb and cervical region. On clinical examination fatty lump was seen in cervical region and muscles had decreased tone. Investigations were done. Magnetic resonance imaging (MRI) showed abnormal mass at C5-C6-C7 level in spinal cord region (A). Initially MRI was taken for cervical region from lateral as well as posterior aspect considering the findings, MRI was repeated to see any involvement in lumbar region (B). No significant abnormality was seen in lumbar region, vertebral bodies showed wedging in the cervical region and fatty mass was seen. The patient was then admitted for this purpose and surgery was planned. Detethering of the spinal cord in cervical region was planned. Myelomeningocele correction was done. Complications of the surgery include cerebrospinal leakage and bladder dysfunction. This patient showed no complications post operatively and was referred to physiotherapy department. The primary goal of the physiotherapist was to prevent secondary complications and to increase the strength of muscles. Special care was taken for cervical region as the patient was post-operative, cervical collar was given and the patient was ambulated on post-operative day 5.

**Figure 1:**
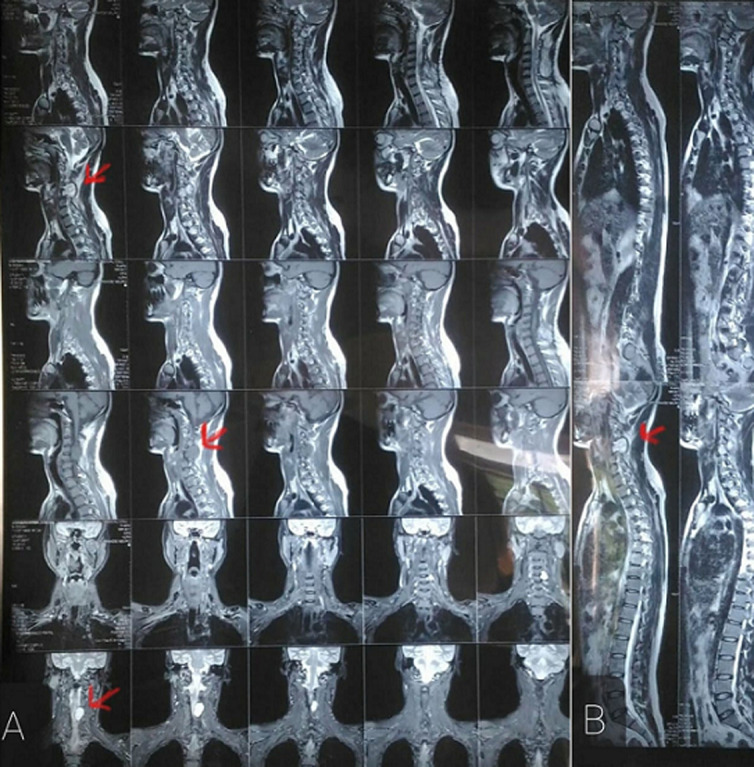
(A,B) tethering of spine in cervical region

